# Study on the Superhydrophobic Properties of Micro/Nano Hole Structure on the Surface of Glass Fiber Reinforced Plastics Based on Femtosecond Laser Etching

**DOI:** 10.3390/nano15040287

**Published:** 2025-02-13

**Authors:** Ji Wang, Guolong Wang, Zhenkai Zhu, Wenwu Zhang

**Affiliations:** 1 Ningbo Institute of Materials Technology & Engineering, Chinese Academy of Sciences, Ningbo 315201, China; wangguolong@nimte.ac.cn (G.W.); zhangwenwu@nimet.ac.cn (W.Z.); 2 Center of Materials Science and Optoelectronics Engineering, University of Chinese Academy of Sciences, Beijing 100049, China; 3 College of Mechanical Engineering, Zhejiang University of Technology, Hangzhou 310014, China

**Keywords:** femtosecond laser processing, super-hydrophobicity, concave hole, GFRP, hydrophobic angle

## Abstract

A method of femtosecond laser multi-pulse grid-like point etching (MP-GPE) was used to prepare glass fiber reinforced plastics with superhydrophobic properties. This article investigates the influence trend of single-pulse energy (5–35 μJ) and etching pulse number (20–100) on the morphology of surface concave holes, including depth and width. Different combinations of process parameters have a modulating effect on the size of the concave hole structure and the ablation of the reinforced plastics. At a single-pulse energy of 25 μJ and 60 pulse numbers, the depth of the concave holes increases to the maximum of approximately 63 μm, and the width of the upper surface of the concave holes is approximately 33 μm. Under these conditions, the maximum water contact angle of 160.6° is obtained, which is consistent with the theoretical calculation results of 161.6°. This is very promising for the power industry to use this material in low-temperature, drag-reducing environments.

## 1. Introduction

Glass fiber reinforced polymer (GFRP) composite materials are extensively used in various fields such as wind power generation, aerospace, transportation, construction, and life and health due to their excellent mechanical properties, lightweight characteristics, and corrosion resistance [1,2,3]. GFRP materials combine the advantages of glass fiber and resin, providing strength and rigidity, making them an indispensable composite material in modern industry. However, the hydrophilicity of GFRP causes it to readily absorb moisture from the air in humid outdoor environments, leading to surface corrosion, low-temperature freezing, and other phenomena that affect material properties and limit its long-term stability and durability. Superhydrophobic surface (SHS) refers to materials where the contact angle between water droplets and water is greater than 150° [4]. This characteristic endows the material with excellent self-cleaning performance, anti-fouling performance, and the power to enhance water droplets to roll off the surface. Super-hydrophobicity enhances the performance in its application fields greatly and extends the service life of the material. Among them, as a widely used material for wind turbine blades, hydrophobic GFRP can effectively solve the problem of blade surface icing and corrosion in cold and humid environments.

Superhydrophobic surfaces (SHSs) have demonstrated significant potential across various fields, including self-cleaning [5,6], anti-icing [7,8,9], oil–water separation [10,11], anti-biofouling [12,13,14], and anti-corrosion [15,16]. Typically, superhydrophobic surfaces enhance the degree of surface roughness through the design of micro/nano structures. These materials are engineered to mimic natural surfaces, such as lotus leaves, featuring micro/nano layered structures that facilitate waterproofing and isolate contact between solids and liquids [17]. Simultaneously, the application of chemical coatings lowers surface energy, in conjunction with micro/nano structures, to decrease the contact angle (CA) between water and solid surfaces, significantly improving the wettability of materials. Sun et al. [18] developed a durable superhydrophobic surface by spraying hydrophobic silica nanoparticles (HSNPs) onto a GFRP surface, achieving a maximum CA of 165.1°. Beyond resisting humid environments, superhydrophobic surfaces can also enhance other specialized application capabilities. For instance, Feng et al. [19] etched microgrooves on the surface of GFRP materials via laser with a thickness two to three orders of magnitude smaller than the wavelength of absorbed sound waves, utilizing the multi-interface scattering effect created by the air layer between water and superhydrophobic micro/nano structures to achieve the characteristic of absorbing incident sound power underwater.

At present, the preparation methods for SHSs mainly include chemical vapor deposition (CVD) [20,21], template methods [22,23], sol–gel methods [24,25], and others. CVD is a technique that deposits materials onto a substrate’s surface through gas-phase reactions. This process typically involves the reaction of gas precursors on the substrate to form a solid thin film, resulting in an SHS. However, this method is not suitable for certain temperature-sensitive substrates, which limits material selection. The template method utilizes pre-prepared templates to create micro/nano structures on surfaces, followed by coating or other treatments to impart superhydrophobic properties. The reliability of repeated production can be affected by the wear and deformation of the template. The sol–gel method involves mixing precursors in solution, forming a polymer film through sol–gel transformation, and finally hardening the substrate’s surface to achieve super-hydrophobicity. While the sol–gel method is simple and efficient, it cannot accurately control film thickness. In summary, these methods have some limitations, such as high cost, specific environmental conditions such as high temperature and vacuum, complex operation, low efficiency, and limitations in metal and material selection.

Pulse laser processing technology, characterized by its ultra-short pulses, offers advantages such as high flexibility, good controllability, and material friendliness [26,27]. In recent years, it has emerged as a promising method for preparing micro/nano surfaces [28,29,30,31]. For instance, Yan et al. [32] proposed a method that combines femtosecond laser element doping with micro/nano structures and cyclic annealing to construct a biomimetic ant nest-like structure dominated by sub-crystalline phases on the surface of metal aluminum alloys, achieving a superhydrophobic CA of 161°. Jiang et al. [33] utilized a femtosecond laser to etch concave pore structures on the surface of aluminum alloy, in conjunction with modified silica nanoparticles and octadecyltrimethylsilane (OTMS) as hydrophobic coatings, achieving superhydrophobic properties with a CA of 165.92°. Wang et al. [34] used a picosecond laser to control the spacing between laser grids and etched dense micro/nano structures on the surface of stainless steel, achieving an SHS with a CA of about 155°, after being left in the air for 24 h. In addition to metals, Luo et al. [35] employed a femtosecond laser to etch microhills and microcolumns on the surface of alumina ceramics with higher hardness. By combining this with high-temperature annealing and adjusting the scanning spacing and height of the microhills, a superhydrophobic effect of 171.7° was attained. However, the heat treatment process takes up to 12 h, which affects the fabrication efficiency.

During laser processing, thermal ablation effects are unavoidable. This processing method, which involves thermal effects, is suitable for materials with high melting points. However, for resin-based materials that are susceptible to thermal decomposition on their surfaces, femtosecond laser processing still presents significant challenges, and there are few reports on related research. This article addresses the issue of excessive ablation on resin-based surfaces by examining the impact of femtosecond laser pulse energy and pulse quantity on the geometric morphology of resin surfaces. Additionally, a grid-like concave hole array microstructure was etched onto the surface of the resin substrate to explore the superhydrophobic properties under various morphologies, achieving a hydrophobic angle of 161.6° without the need for additional coating or high-temperature annealing treatment.

## 2. Materials and Methods

### 2.1. Method and Experimental Setup

We proposed a method of femtosecond laser multi-pulse grid-like point etching (MP-GSE) to prepare an SHS on GFRP.

**Figure 1:**
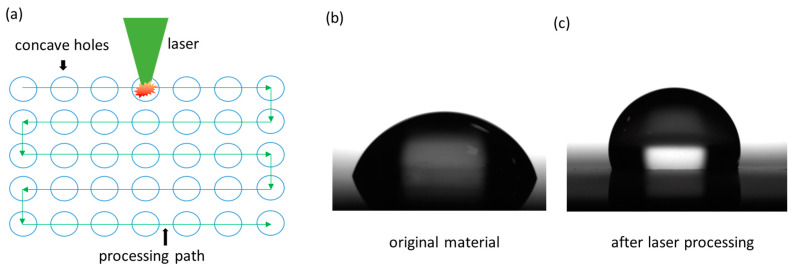
Schematic diagram of the method from hydrophilicity to super-hydrophobicity of samples: (**a**) path design for concave hole array etching by focusing femtosecond laser; (**b**) hydrophilic droplet state on the original sample surface; (**c**) hydrophobic droplet state after laser treatment.

The experiment used a femtosecond laser emitting in the green wavelength band to etch a concave hole array on the surface of this material along a predetermined path. A femtosecond laser beam with a diameter of approximately 3 mm was incident on a field lens with a focal length of 58.5 mm to form a focused spot with a diameter of approximately 25 μm. The samples to be etched were placed on a three-axis adjustable electrically controlled motion platform with a working range of 200 mm (X) × 200 mm (Y) × 20 mm (Z). The focused light spot fell on the upper surface of the sample via adjusting the position of the Z-axis. The motion speed of the motion table was 1 mm/s (maximum speed is 20 mm/s), with a resolution of 1 μm on the X/Y axis. The experiment adopted a mobile single-point processing method to achieve the preparation of large-scale concave hole structures. As shown in Figure 1, the motion trajectory is similar to a “bow” shape. Once the preset number of pulses was emitted, the X-axis continued to move at a speed of 1 mm/s to the next position for etching the next concave hole. In the experiment, the distance between points was set to 40 μm. The laser processing parameters used in the experiment are shown in Table 1.

After laser preparation, the samples were surface modified with silane solution. Figure 1b,c show the surface droplet states of the original sample and the sample prepared in this experiment, respectively. In its original state, the sample exhibited hydrophilicity, with a contact angle CA of 74.6°. After laser etching, the surface structure morphology is affected, and the roughness is increased. After silane modification to reduce surface energy, the hydrophobic angle is significantly improved.

Firstly, the samples were placed in a beaker filled with ethanol after laser processing. The samples were sonicated using a water bath method for 10 min. After drying, the samples were immersed in a 1% solution of trimethoxy (1H, 1H, 2H, 2H heptafluorodecyl) silane butyl acetate for surface chemical modification. Then, after soaking for 3 h, the samples were removed and placed in a high-temperature chamber at 100 °C. After 1 h, we turned off the power and let it cool naturally.

### 2.2. Materials

The material used in this research is glass fiber reinforced polymer, with a size of 20 mm × 20 mm × 1 mm, purchased from Shanghai Yuyu Rubber and Plastic Products Co., Ltd. (Shanghai, China). Its basic material is epoxy-resin, which is woven with diameter of 10 μm glass fibers in a grid-like pattern to form the reinforcing material. The ethanol (C_2_H_5_OH) with 98% purity and trimethoxy (1H,1H,2H,2H heptadecafluorodecyl) silane with 98% purity was purchased from Shanghai Aladdin Biochemical Co., Ltd. (Shanghai, China). The butyl acetate (CH_3_COO(CH_2_)_3_CH_3_) with 99% purity was purchased from Sigma-Aldrich Trading Co., Ltd. (St. Louis, MO, USA). Trimethoxy (1H, 1H, 2H, 2H heptafluorodecyl) silane was mixed with butyl acetate in a beaker at a volume ratio of 1:99.

### 2.3. Instruments and Data Processing

The model of the femtosecond laser (YSL photonics Co., Ltd., Wuhan, China) used in the experiment is FemtoYL. A scanning electron microscope (SEM) (model MAFNA, TESCAN, Brno, Czech Republic), another scanning electron microscope (model S4800, Hitachi, Hitachi city, Tokyo), and a laser confocal scanning microscope (LCSM) (model VK-X200K, ZEISS, Oberkochen, Germany) were used to observe the surface structures of the samples. A contact-angle-measuring instrument (model SCI3000F, Global Hengda Technology Co., Ltd., Beijing, China) was used to characterize the hydrophobic properties of the samples.

The depth and width results of the concave holes were calculated from the average and standard deviation of five sets of raw data. The CA results were calculated from the average and standard deviation of four sets of measurement results. The data following the ± values in this text represent the standard deviation.

## 3. Results

### 3.1. Influence of Pulse Energy

#### 3.1.1. Influence of Pulse Energy on Surface Microstructure

**Figure 2:**
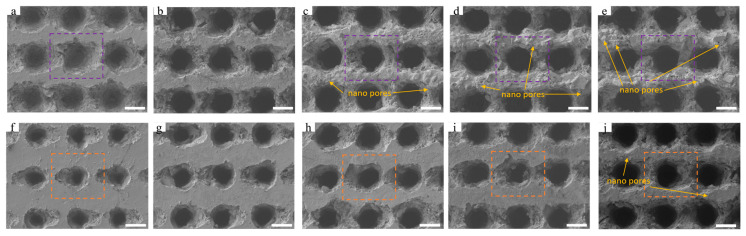
SEM images of concave hole array surfaces under different laser energies: (**a**–**e**) are SEM images of concave hole arrays formed with single-pulse energies of 5, 10, 15, 20, and 25 μJ, respectively, when the laser outputs 60 pulses at each concave hole position; (**f**–**i**) are SEM images of concave hole arrays formed with single-pulse energies of 5, 10, 15, 20, and 25 μJ, respectively, when the laser outputs 40 pulses at each concave hole position. The scales are 20 μm in Figure 2 (**a**–**j**). The yellow arrow indicates that laser ablation is accompanied by the generation of nano pores.

The surface layer of GFRP material is composed of a resin layer. The laser removal process of resin depends on the high energy density of the laser beam. The laser selectively absorbs and rapidly heats the resin to its decomposition or gasification temperature, facilitating its removal. By using the MP-GSE method, large-scale concave holes have been successfully etched. Firstly, we investigate the influence of laser pulse energy on the geometric morphology of concave holes. As the single-pulse energy changes from 5 μJ to 25 μJ, the morphologies of the concave holes are illustrated in Figure 2. The laser pulse frequency is set at 100 kHz. Figure 2a–e shows the results of processing each concave hole with 60 pulses at single-pulse energies of 5 μJ, 10 μJ, 15 μJ, 20 μJ, and 25 μJ, respectively. Figure 2f–j displays the results of 40 pulses, with single-pulse energies of 5 μJ, 10 μJ, 15 μJ, 20 μJ, and 25 μJ, respectively. For energy below 10 µJ, an increase in the number of pulses leads to a significant increase in the surface diameter of the concave hole. When the energy is 10 μJ, the average surface diameter of the concave hole increases by about 6.4 μm. However, at energies (15 μJ, 20 μJ, 25 μJ), as the number of pulses increases from 40 to 60, the average increase in the diameter of the concave hole surface is halved to 3.4 μm, 3.1 μm, and 2.6 μm, respectively. That is to say, when the energy exceeds 10 µJ, as the number of pulses rises from 40 to 60, the surface diameter of the concave holes remains relatively stable. 

Concerning the grid-like surface between concave holes, regardless of the increase in energy (from 5 μJ to 25 μJ) and the number of pulses (from 40 to 60), the intensity of laser’s effect on this area gradually intensifies. Initially, the grid-like surface is flat, with a spacing of approximately 4.3 µm (at 60 pulses) and 8.2 µm (at 40 pulses). As energy increases, the thermal ablation effect of the laser on the resin surface is enhanced. The girder structure between adjacent concave holes is progressively compressed, and the surface flatness cannot be maintained. As shown in Figure 2f–i, with fewer than 40 pulses, there are no nanopores or remelted particles caused by laser ablation on the surface of the area. With 40 pulses and a single-pulse energy of 25 µJ, noticeable edge fractures and a few nanopores are present on the surface in this region. Figure 2a illustrates that when the number of pulses reaches 60 and the energy is 5 µJ, the surface of the girder structure between adjacent concave holes remains smooth, similar to that shown in Figure 2f–i. The material between the concave holes undergoes remelting under repeated pulse action with a pulse interval of 10 µs. As the energy increases from 10 µJ to 25 µJ, more nanopores and remelted particles appear, and the remelted area gradually expands (Figure 2b–e). This leads to a significant increase in the roughness of samples.

**Figure 3:**
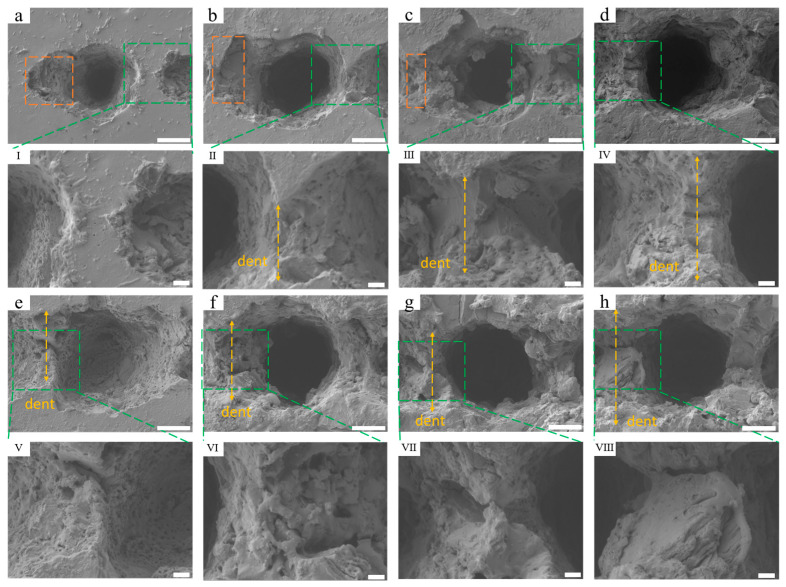
SEM images of a single concave hole and its edges: (**a**–**d**) are the individual concave hole morphologies in the orange dashed box area in Figure 2. The orange box represents the trailing effect caused by the last pulse; (**e**–**h**) are the individual concave hole morphologies in the purple dashed box area in Figure 2; (**I**–**IV**) are enlarged partial views of the green dashed boxes in (**a**–**h**). The scales of (**a**–**h**) are 10 μm. The scales of (**I**–**VIII**) are 2 μm.

Figure 3 illustrates the microstructure of a single concave hole and the girder structure between concave holes is depicted in Figure 2. A shallow groove is present on one side of the concave hole. This occurs due to an asynchronization issue between the pulse shutdown signal received by the laser and the initial movement signal from the motion table, leading to a trailing effect of the last pulse, as indicated by the yellow dashed box in Figure 3a–c. Furthermore, as the energy increases, the flat girder structure between the concave holes is significantly eroded by laser pulses, resulting in the formation of depressions, as shown in Figure 3II–VIII). The laser energy threshold at which the contour edges of adjacent concave holes start to merge varies depending on the number of pulses. When the number of pulses reaches 40 and the energy exceeds 15 μJ, the contour edges of adjacent concave holes gradually merge. As the energy rises from 15 μJ to 25 μJ, the length of the recess increases from approximately 9.4 μm to 15.4 μm with 40 etching pulses. When the number of pulses is 60 and the energy surpasses 10 μJ, the contour edges of adjacent concave holes gradually connect, and the length of the concave hole increases from about 8.7 μm (Figure 3e) to 14.3 μm (Figure 3h). Additionally, we observed that the inclination angle of the inner wall of the concave hole increases with rising energy, resulting in the girder structure between the concave holes being eroded into a shape of peak, indicating an expansion of the volume inside the concave holes. Concurrently, the girder structure is accompanied by holes or slits with sizes ranging from sub-micron to 2 microns. As an air chamber, the concave holes store air, which is crucial for the generation of super-hydrophobicity.

#### 3.1.2. Ablation Effect of Excessive Energy on Surface Structure

**Figure 4:**
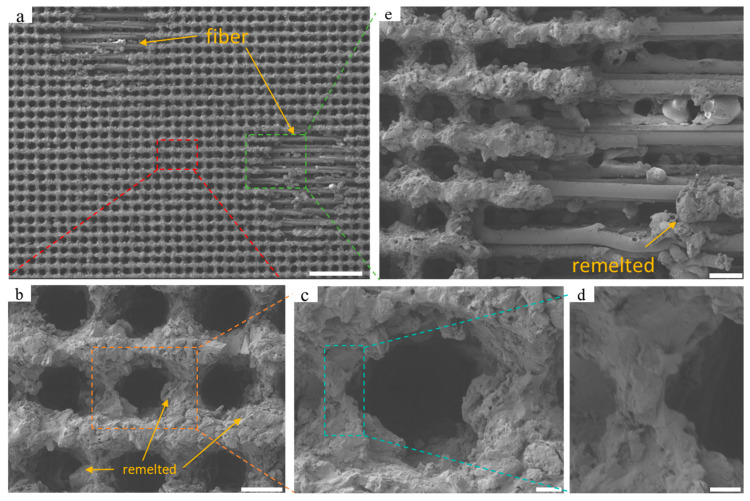
SEM images of the sample surface at high energy of 30 μJ: (**a**) surface morphology within the range of 1.37 mm × 1.02 mm, with a scale of 200 μm. The yellow arrow refers to the remelted particles and exposed fibers; (**b**) microstructure of a 3 × 3 array of concave holes within the red dashed box in (**a**), with a scale of 20 μm. The yellow arrow refers to the remelted particles; (**c**) local microstructure of a single concave hole within the orange box in (**b**), with a scale of 5 μm; (**d**) local microstructure of beams between adjacent concave holes within the blue box in (**c**), with a scale of 2 μm; (**e**) microstructure of exposed glass fibers on the surface within the green box in (**a**), with a scale of 25 μm. The yellow arrow refers to the remelted particles.

**Figure 5:**
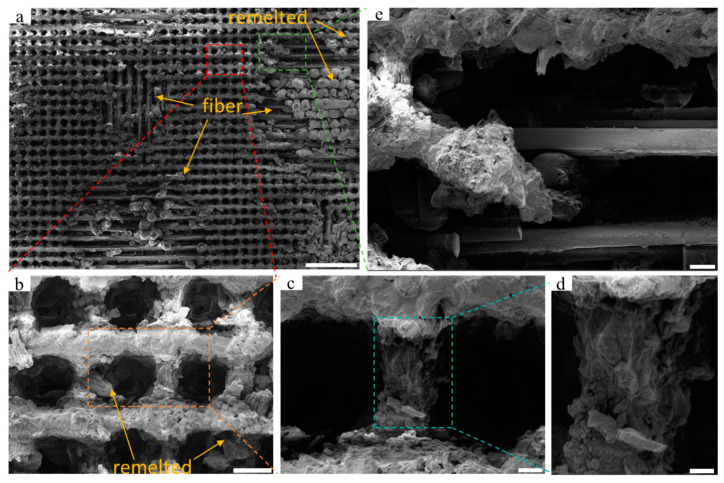
SEM images of the sample surface at an energy of 35 μJ: (**a**) surface morphology within the range of 530 μm × 480 μm, with a scale of 200 μm. The yellow arrow refers to the remelted particles and exposed fibers; (**b**) microstructure of a 3 × 3 array of concave holes within the red dashed box in (**a**), with a scale of 20 μm. The yellow arrow refers to the remelted particles; (**c**) microstructure of a single local concave hole within the orange box in (**b**), with a scale of 5 μm; (**d**) microstructure of beams between adjacent local concave holes within the blue box in (**c**), with a scale of 2 μm; (**e**) microstructure of exposed glass fibers on the surface within the green box in (**a**), with a scale of 10 μm.

When the energy further increases to 30 μJ, excessive energy can lead to significant erosion, as the resin surface is susceptible to decomposition when heated. When the pulse energy is set at 60, the single-pulse energy is further raised to 30 μJ and 35 μJ, with the processing results illustrated in Figure 4 and Figure 5, respectively. At a pulse energy of 60 and single-pulse energy of 30 μJ, two exposed areas of glass fiber measuring approximately 380 μm × 380 μm within the processing area of 1.37 mm × 1.03 mm are observed (see Figure 4). At this stage, the girder structure between the concave holes experienced severe erosion, resulting in a more complex microscale granular structure on the surface, leading to a significant increase in surface roughness. Partially removed resin is scattered on the inner walls of the concave holes or on exposed fiber surfaces, and as the temperature decreases, these resins form remelted particles. This phenomenon suggests that lower pulse energy still induces resin decomposition and ablation, thereby impacting the overall processing outcome.

As the single-pulse energy was further increased to 35 μJ, the ablation effect became more pronounced, with at least five fiber-exposed areas appearing, and the size of the exposed areas increased to approximately 530 μm × 480 μm. At this point, not only the horizontally arranged glass fibers are exposed, but also the vertically arranged fibers begin to appear, with a diameter of about 1 μm for the exposed glass fibers. The rise in laser energy results in a significant increase in resin particles deposited after thermal decomposition. Then, it creates sub-nanometer level rough structures in the girder structure area without direct laser irradiation.

**Figure 6:**
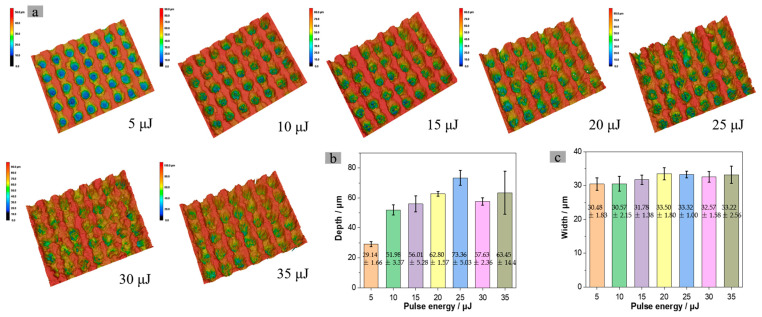
Depth and width of concave holes at different energies: (**a**) SCM three-dimensional morphology of concave hole array with 60 pulses and different single-pulse energies; (**b**) depth histogram of concave holes; (**c**) bar chart showing the width of the upper surface of the concave hole.

The three-dimensional morphology of the concave hole array was measured to observe the depth of the concave holes and the width of the upper surface. The results are shown in Figure 6 (measuring five concave holes, taking the average and variance). When the energy increases from 5μJ to 10 μJ, the average depth of the concave hole doubles, from 29.14 ± 1.66 μm to 51.98 ± 3.37 μm. As the energy continues to increase to 25 μJ, the average depth of the concave holes also increases, measuring 56.01 ± 5.28 μm, 62.80 ± 1.57 μm, and 73.36 ± 5.03 μm, respectively. However, the depth of the concave holes does not increase indefinitely with increasing energy. When the energy exceeds 30 μJ, the surface structure begins to degrade, with depths of 57.63 ± 2.36 μm and 63.45 ± 14.4 μm. Moreover, the depth of the concave holes in the array fluctuates greatly. The threshold for resin pyrolysis is relatively low (usually with a melting point of approximately 150–200 °C). Even at lower energy (5 μJ), the laser can still excite enough heat longitudinally to decompose and remove materials, resulting in a significant increase in depth. However, due to excessive energy (reaching 30 μJ), the heat-affected area of the laser beam in the transverse direction increases invisibly. The surrounding grid-like structure begins to undergo thermal decomposition and collapse under the influence of heat (at pulse energy of 30 μJ and 35 μJ). A portion of the resin particles that undergo thermal decomposition are deposited into the concave pores along the slope, resulting in a decrease in depth and stability of the formed structure (Figure 6b). In Figure 6a, as the energy increases from 5 μJ to 35 μJ, and the average surface roughness measurement results are 8.3 μm, 14.1 μm, 16.7 μm, 17.9 μm, 18.5 μm, 14.7 μm, and 16.1 μm, respectively (the measurement areas are all rectangles of 100 μm × 100 μm, and the average value is taken for the five measurement areas). It can be observed that the trend in roughness changes is consistent with the depth.

In contrast, the width of the upper surface of the concave hole does not change significantly with increasing energy. Their average widths on the upper surface are 30.48 ± 1.83 μm, 30.57 ± 2.15 μm, 31.78 ± 1.38 μm, 33.50 ± 1.80 μm, 33.32 ± 1.00 μm, 32.57 ± 1.58 μm, and 33.22 ± 2.56 μm, respectively. The width exhibits a slight increase. The increased laser energy causes the surface temperature of the material to rise, allowing heat conduction to penetrate the interior of materials. Although this thermal diffusion elevates the temperature of the material in the near-surface area, it may not immediately lead to an increase in the width of the concave hole, as most energy remains concentrated at the laser focus, resulting in more material being removed from a smaller area. In addition, under high-energy pulse conditions (≥25 μJ), the heating of the beam forms a sub-micron level rough surface or collapse, which to some extent reduces the width value.

#### 3.1.3. Effect of Laser Energy on Wettability of the Concave Hole Surface

**Figure 7:**
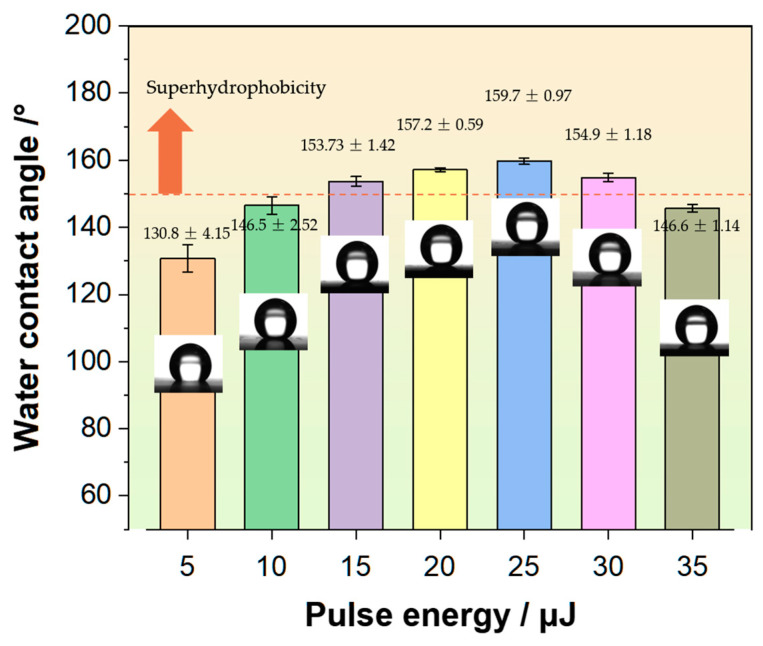
Contact angle values and surface droplet states under different laser pulse energies.

Figure 7 illustrates the CA of the sample surface at laser single-pulse energies of 5 μJ, 10 μJ, 15 μJ, 20 μJ, 25 μJ, 30 μJ, and 35 μJ, with 60 pulses. The CA is 130.8 ± 4.15°, 146.5 ± 2.52°, 153.7 ± 1.42°, 157.2 ± 0.59°, 159.7 ± 0.97°, 154.9 ± 1.18°, and 146.6 ± 1.14°, respectively. As the laser pulse energy increases, the surface hydrophobicity initially rises and then decreases, with the highest hydrophobicity observed at 25 μJ. After calculation, when the pulse energy is 5 μJ, the percentage β is 45.6% (5 μJ) for the combined areas of the upper surfaces of concave holes and rough-structured surfaces to the total surface area, while the remaining regions are flat surface exhibiting strong adhesion. When the laser pulse energy is increased to 15 μJ, the average CA reaches 153.73°, meeting the superhydrophobic standard. The surface roughness of the girder structure under this parameter increases, resulting in β being 90.3% at 15 μJ. At the average laser power of 20 μJ (β is 93.6%) and 25 μJ (β is 100%), the CA further improves, with the maximum CA exceeding 160°, reaching 160.6°. However, when the energy increases to 30 μJ and 35 μJ, the morphology of the concave hole array on the surface begins to deteriorate, leading to a decreasing trend in the surface contact angle. As energy increases, the exposed area of fibers grows, disrupting the periodic array micro/nano structure on the surface. At 35 μJ, the CA falls below the superhydrophobic standard value of 150°. The surface micro/nano structure is crucial for achieving super-hydrophobicity.

### 3.2. Influence of Pulse Number

#### 3.2.1. Influence of Laser Pulse Number on Surface Microstructure

**Figure 8:**
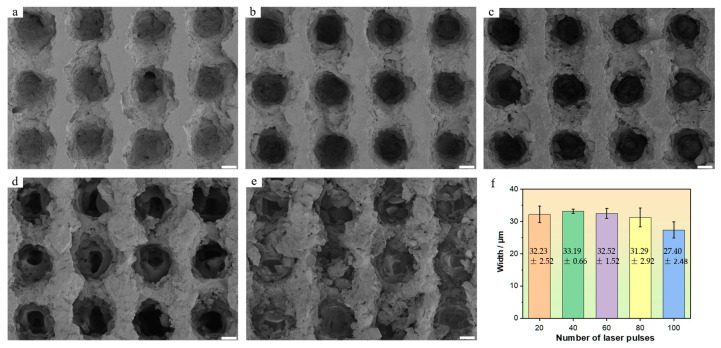
SEM morphology of the sample surface obtained by etching with different pulse numbers at single-pulse energy of 20 μJ: (**a**) 20; (**b**) 40; (**c**) 60; (**d**) 80; (**e**) 100; (**f**) bar chart showing the upper surface width of the concave hole. The scales of (**a**–**e**) are 10 μm.

**Figure 9:**
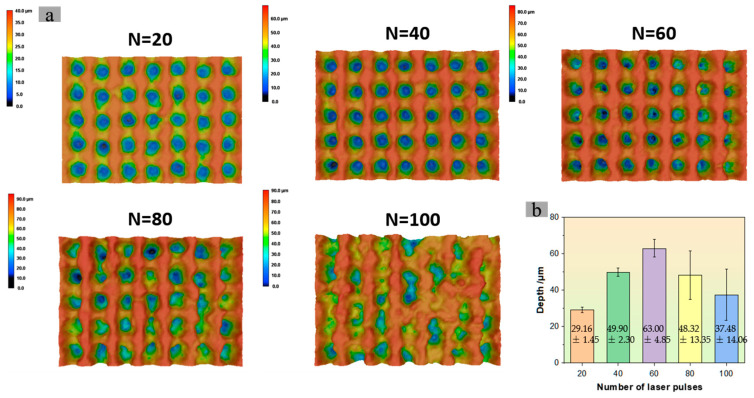
Three-dimensional morphology under LCSM: (**a**) 3D morphology of samples obtained by etching with different pulse numbers at single-pulse energy of 20 μJ, with pulse numbers of 20, 40, 60, 80, and 100; (**b**) measurement results of concave hole depth.

The energy of a single laser pulse is 20 μJ, with a frequency repetition rate of 100 kHz. By modulating the pulse irradiation frequency at the etching point, five samples were obtained, and their SEM surfaces and 3D morphologies are shown in Figure 8 and Figure 9. Among them, Figure 8a–e show the effects when using 20, 40, 60, 80, and 100 pulses respectively.

The concave hole exhibits a tailing phenomenon along the direction of motion. Edge connections form between adjacent concave holes. As the number of etching pulses increases, the middle girder structure gradually becomes rougher, and the hole depth intensifies. A small amount of nanoscale particle structure begins to appear on the girder surface. When the number of pulses reaches 80, the girder structure begins to collapse on a large scale, becoming a dispersed weathered-stone shape. The surface of this structure is covered with micro/nano particles. The materials removed from the gradually collapsing beam structure cling to the inner wall of the concave hole, hindering the etching effect of subsequent pulses on depth. After further increasing to 100 pulses, the degree of the girder structure collapse intensifies. The removed materials slide down the slope and accumulate in the concave hole. The upper surface width of the concave hole does not change significantly, avoiding excessive etching that would cause girder structure collapse. However, once the pulse count becomes excessive, the girder structure collapses, leading to a corresponding decrease in the upper surface width of the concave hole. The results indicated that with varying pulse numbers, the widths are 32.23 ± 2.52 μm, 33.19 ± 0.66 μm, 32.52 ± 1.52 μm, 31.29 ± 2.92 μm, and 27.40 ± 2.48 μm, respectively. Notably, when the pulse count is between 20 and 60, the width remains relatively stable at a considerable level. Upon reaching 80 pulses, there is a slight decrease in width accompanied by a significant increase in fluctuation amplitude. At 100 pulses, the width further decreases, and the fluctuation amplitude also becomes significant.

The particle sizes formed by removing the weathered-stone structure vary, and the filling degree of the concave holes is also uneven. This leads to severe fluctuations in the overall geometric morphology of the concave holes in the sample, especially in the depth parameter, when the number of pulses is too large. As the number of pulses increases, the average depth of the concave holes is 29.16 ± 1.45 μm, 49.90 ± 2.30 μm, 63.00 ± 4.85 μm, 48.32 ± 13.35 μm, and 37.48 ± 14.06 μm, respectively. Among them, when the number of pulses is 60, the depth is the maximum. In Figure 9a, as the pulse number increases from 20 to 100, the average surface roughness measurement results are 8.4 μm, 12.6 μm, 15.9 μm, 14.5 μm, and 11.5 μm, respectively (the measurement areas are all rectangles of 100 μm × 100 μm m, and the average value is taken for the five measurement areas). It can be observed that the trend in roughness changes is consistent with the depth, with the maximum occurring at 60 pulses.

#### 3.2.2. Effect of Laser Pulse Number on Wettability of the Concave Hole Surface

**Figure 10:**
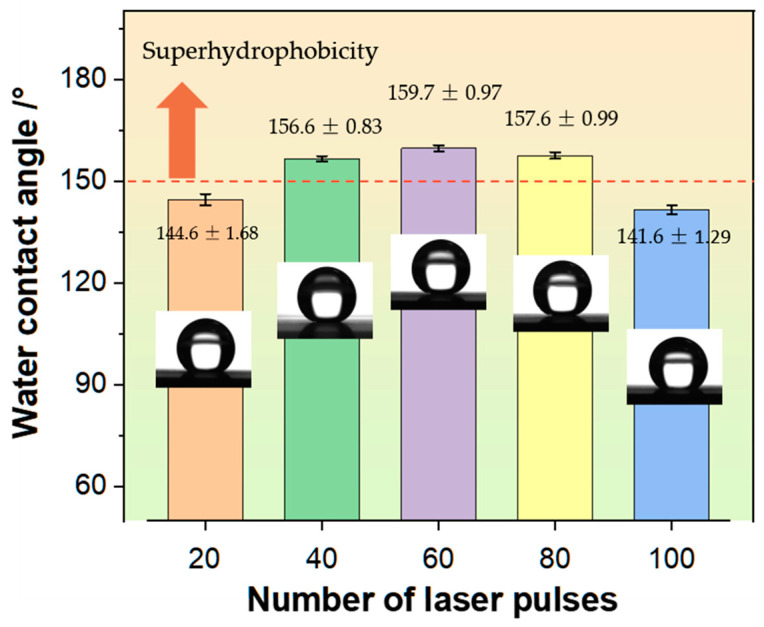
Contact angle data and surface droplet states under different laser pulse number.

The CA of the prepared GFRP materials’ surface is shown in Figure 10 under the variation of pulse number from 20 to 100. When the pulse number is 20, the large flat surface area exhibits strong adhesion, with an average CA of 144.6 ± 1.68°, indicating that it has not reached a superhydrophobic state. At pulse 40, despite the relatively large flat surface area, the depth of the concave hole increases, yielding a hydrophobic angle of 156.6 ± 0.83°. At 60 single point pulses, the surface exhibits the strongest hydrophobicity, with a contact angle of 159.7 ± 0.97°. At this stage, the depth of the concave holes on the surface is at its maximum, forming a rough micro/nano composite structure. The hydrophobic angle peaks at 160.4°. At pulse numbers 80 and 100, the surface contact angles are 157.6 ± 0.99° and 141.6 ± 1.29°, respectively. Compared to the optimal state, hydrophobic performance declines due to the expansion of the laser heat-affected zone, which reduces the depth of the concave holes. By increasing the irradiation time, surface roughness and pit depth can be enhanced within a certain range; however, excessive irradiation can lead to significant laser thermal effects, damaging the surface array structure and consequently affecting surface hydrophobicity. This aligns with the influence law of pulse energy.

## 4. Discussion

From the analysis of the influence of pulse energy and pulse quantity on the 3D morphology and hydrophobicity of concave holes, it is found that the width and depth of concave holes are the main factors affecting surface hydrophobicity. According to the Cassie–Baxter theory, in superhydrophobic conditions, droplets are suspended on the micro/nano structures of the sample surface. Then, the contact angle of the droplet is represented by Equation (1) [36,37], which considers the air contained by micro/nano structures:

(1) $$\cos\theta =f_{s-l}\times \cos\theta_{s-l}+f_{g-l}\times \cos\theta_{g-l},$$

where θ is the equilibrium contact angle, $\theta_{s-l}$ is the intrinsic contact angles of the droplet when in contact with an ideal smooth solid surface, and $\theta_{g-l}$ is the intrinsic contact angles of the droplet when in contact with an ideal smooth solid surface and a gas. Using $f_{s-l}$ and $f_{g-l}$ as the area fractions of the solid–liquid contact surface and the liquid gas contact surface in the entire apparent contact area,

(2) $$f_{s-l}+f_{g-l}=1$$

Then, Equation (1) can be rewritten [38] as:

(3) $$\cos\theta =f_{s-l}\times \cos\theta_{s-l}+f_{s-l}-1,$$

According to Equation (3), the smaller the solid–liquid contact fraction $f_{s-l}$, the more gas there is at the contact interface, and the larger the contact angle of the droplet on the surface. Therefore, in the case where the wettability of the material itself is difficult to change, designing surface microstructures that can store more gas and reduce the solid–liquid contact area is an important means to improve the contact angle and enhance surface hydrophobicity. Droplets stay on microstructures composed of surface concave hole arrays, and their contact state mainly depends on the surface energy and surface microstructure of the solid surface [39,40]. Solid surfaces with lower surface energy and surface microstructures with deep structures can effectively prevent liquid droplets from entering the interior, allowing for the storage of a certain amount of air in the microstructure, thereby forming a stable Cassie–Baxter state [41,42,43]. The concave hole array structure prepared by our research institute can effectively form this type of cavity, isolating the contact between liquid droplets and solid surfaces. Furthermore, the combination of microscale pores and nanoscale roughness in the girder structure enhances the surface hydrophobicity.

In this experiment, we assume that the contact area between the upper part of the droplet and the air is S_1_, and the contact surface area between the droplet and the microcavity formed by the concave hole array is S_2_. The contact area between the droplet and the surface of the concave hole array is S_3_. The mathematical relationship between them is shown in Equations (4)–(6).

(4) $$S_{1}=4\pi R^{2}-2\pi R^{2}\times \frac{(\sin\theta {)}^{2}}{\cos\theta },$$

(5) $$S_{2}={\sum_{i=1}^{N}\pi \left(\frac{D}{2}\right)}^{2}$$

(6) $$S_{2}+S_{3}=\pi r^{2}$$

Here, R is the radius of the spherical droplet, r the radius of the circular area formed by the droplet in contact with the sample surface, N is the number of concave holes covered in the circular area formed by the droplet in contact with the sample surface, and D is the width of the upper surface of the concave hole.

The proportion of solid–liquid area fraction *f_s_* is as follows:

(7) $$f_{s-l}=\frac{S_{1}}{S_{1}+S_{2}+S_{3}}\times 100\%,$$

Formula (7) shows that the proportion of solid–liquid surface contact is negatively correlated with the upper surface width of the concave hole. The larger the concave hole, the smaller the proportion of solid–liquid area, which is not conducive to obtaining a superhydrophobic state. When the proportion of solid–liquid contact surface exceeds 6.0%, the theoretical hydrophobic angle is less than 150°. If the volume of the droplet is 6 μL, after calculation, the solid–liquid area ratio $f_{s-l}$ is approximately 2.28% and $f_{g-l}$ is approximately 97.72%. The large amount of air trapped in the concave pores of the microstructure will greatly increase the gas/liquid interface, effectively preventing liquid from penetrating into the pores. When the intrinsic contact angle of the original sample was measured to be 74.6° (as shown in Figure 1c), the theoretical contact angle θ was calculated to be 161.6° according to Equation (3). This matches the maximum hydrophobic angle of 160.6° (60 pulses and 25 μJ, as shown in Figure 7 and Figure 8) obtained in this study.

**Figure 11:**
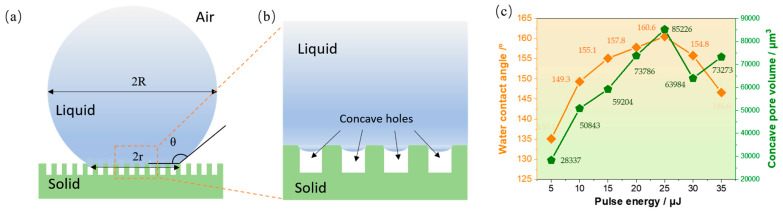
Analysis of superhydrophobic mechanism: (**a**) schematic diagram of superhydrophobic droplets based on microporous structure; (**b**) schematic diagram of droplet immersion in concave pore structure in the dashed box of (**a**); (**c**) relationship between concave cavity volume and measured average hydrophobic angle, with different energy values under 60 pulses.

The rough features and the shape of their edges can affect the contact angle [44]. The small curvature of depressions, fibers, or columns can also alter the wetting behavior of liquids [45]. In practice, the opening width of the concave hole is large, but in the case of shallow depth, there is not enough gas to provide a supporting force for the droplet, and the droplet will sink into a part of the concave hole, as shown in Figure 11b. For superhydrophobic surfaces with a Cassie impregnation wetting state, water can wet rough solid surfaces, but will not completely impregnate surface micro/nano structures [46,47]. Because the accumulation of pressure in holes or between short distances, microparticles can prevent liquid from penetrating into porous structures, which is generally typically attributed to the influence of micro/nano structures that create rough surfaces [48,49,50].

The modification of the Cassie–Baxter equation (Equation (3)) takes into account the roughness of the solid surface, as follows [51]:

(8) $$\cos\theta =m*f_{s-l}\times \cos\theta_{s-l}+1-f_{s-l}$$

Here, m is the roughness factor, which is the ratio of the actual area of the concave pore structure in contact with the droplet to the projected area. Thus, the value is always greater than 1. According to Equation (8), for hydrophobic surfaces (>90°), the CA on rough surfaces increases as the solid–liquid interface decreases. The surface concave pore structure with air chambers will amplify the wettability of the material [52,53]. This is the reason why this study uses femtosecond laser single-pulse point etching to form surface concave hole structures to achieve superhydrophobic properties.

Of course, in addition to the width dimension, the hydrophobic angle is also related to the volume of the air chamber. Therefore, a comprehensive analysis is needed.

Figure 11c intuitively shows the relationship between the experimentally obtained surface wettability and the total volume of the gas chamber formed by the concave hole array covering the bottom of the droplet at different energies. When the energy increases from 5 to 25 μJ, there is a positive correlation between the changing trend in hydrophobic angle and the total volume of the gas chamber. At 25 μJ, a maximum hydrophobic angle of 160.6° is obtained, and the total volume of the concave pore chamber covered by the bottom of the droplet is also the largest, 85,226 μm^3^. When the energy reaches 30 μJ, excessive energy causes fiber exposure in some surface areas (Figure 4) and a decrease in concave pore depth (Figure 6b), resulting in a significant decrease in volume to 63,984 μm^3^. At this point, the surface hydrophobicity angle decreases and reaches 154.8 °. As the energy further increases to 35 μJ, fiber exposure and fracture appear in most areas of the surface (Figure 5). At this point, although the volume has increased to 73,273 μm^3^, the depth fluctuation of the concave hole is abnormally severe (Figure 6b), and the root mean square difference suddenly increases from 2.36 μm (30 μJ) to 14.4 μm. Overall, the surface hydrophobicity angle continues to decrease and reaches 146.6°.

## 5. Conclusions

In this article, we proposed a multi-pulse grid-like point etching (MP-GPE) method based on femtosecond laser for etching concave hole microstructures on the surface of glass fiber reinforced plastics. The effects of femtosecond laser pulse energy (5–35 μJ) and the number of pulses (20–100) at a single point on the geometric morphology and contact angle of the resin surface was investigated. This MP-GPE method is straightforward and effective, eliminating the need for additional complex chemical coatings or high-temperature treatment techniques. By adjusting the laser process parameters, the dimensions of the concave hole structure can be fine-tuned, including depth and width. When the pulse energy increases from 5 to 25 μJ, the average depth of the concave hole increases from 29.14 μm to 73.76 μm, while the average CA increases from 130.8° to 159.7°. At a pulse energy of 30 μJ, the depth of the concave hole decreases to 57 μm and the CA decreases to 154.9°. The width of the concave hole is maintained between 30 and 33 μm. Additionally, this research addressed the challenges posed by thermal ablation effects during surface processing of resin-based materials. The phenomenon of glass fiber exposure and fracture is suppressed when the pulse energy is less than 30 μJ. As the number of pulses increases from 20 to 100, both the depth of the concave hole and the CA initially increase and then decrease. The depth reaches 63 μm at the pulse number of 60. The structure around the concave hole collapses severely at a pulse number of 100, causing the concave hole to become buried and the CA to decrease to 146°.

Finally, an SHS with a maximum CA of 160.6° was achieved under a single-pulse energy of 25 μJ and etching with 60 multi-pulses. Moreover, we proposed a predictive model for contact angle, and the theoretical calculation results obtained from the model are highly consistent with the experimental values. These findings establish a foundation for the advancement of efficient, passive hydrophobic, and self-cleaning technologies, which are expected to be applied in high-performance materials used in fields such as energy, aerospace, automotive, and construction. Especially, as the core material for wind turbine blades and building exterior walls, this study will promote the application and development of GFRP in improving wind power generation and solar thermal deicing panels. Of course, this study still has certain limitations, and further exploration is needed in the future to investigate the application of this technology in the field of superhydrophobic anti-icing and its long-term durability.

## Figures and Tables

**Table 1:** Laser processing parameters.

Physical Quantity	Data	Physical Quantity	Data
wavelength/nm	516	M^2^	x: 1.09; y: 1.15
repetition frequency/kHz	100	pulse width/fs	373
pulse energy/μJ	5–35	pulse number	20–100
scanning speed/mm/s	1	scanning spacing/μm	40

## Data Availability

The original contributions presented in the study are included in the article; further inquiries can be directed to the corresponding author.
